# Supplementary material for: An Injectable Hyaluronan–Methylcellulose (HAMC) Hydrogel Combined with Wharton’s Jelly-Derived Mesenchymal Stromal Cells (WJ-MSCs) Promotes Degenerative Disc Repair
Source: Int J Mol Sci. 2020 Oct 7;21(19):7391. doi: 10.3390/ijms21197391 (PMC7582266; doi:10.3390/ijms21197391)
Supplement: Supplementary file 1 [file ijms-21-07391-s001.pdf]

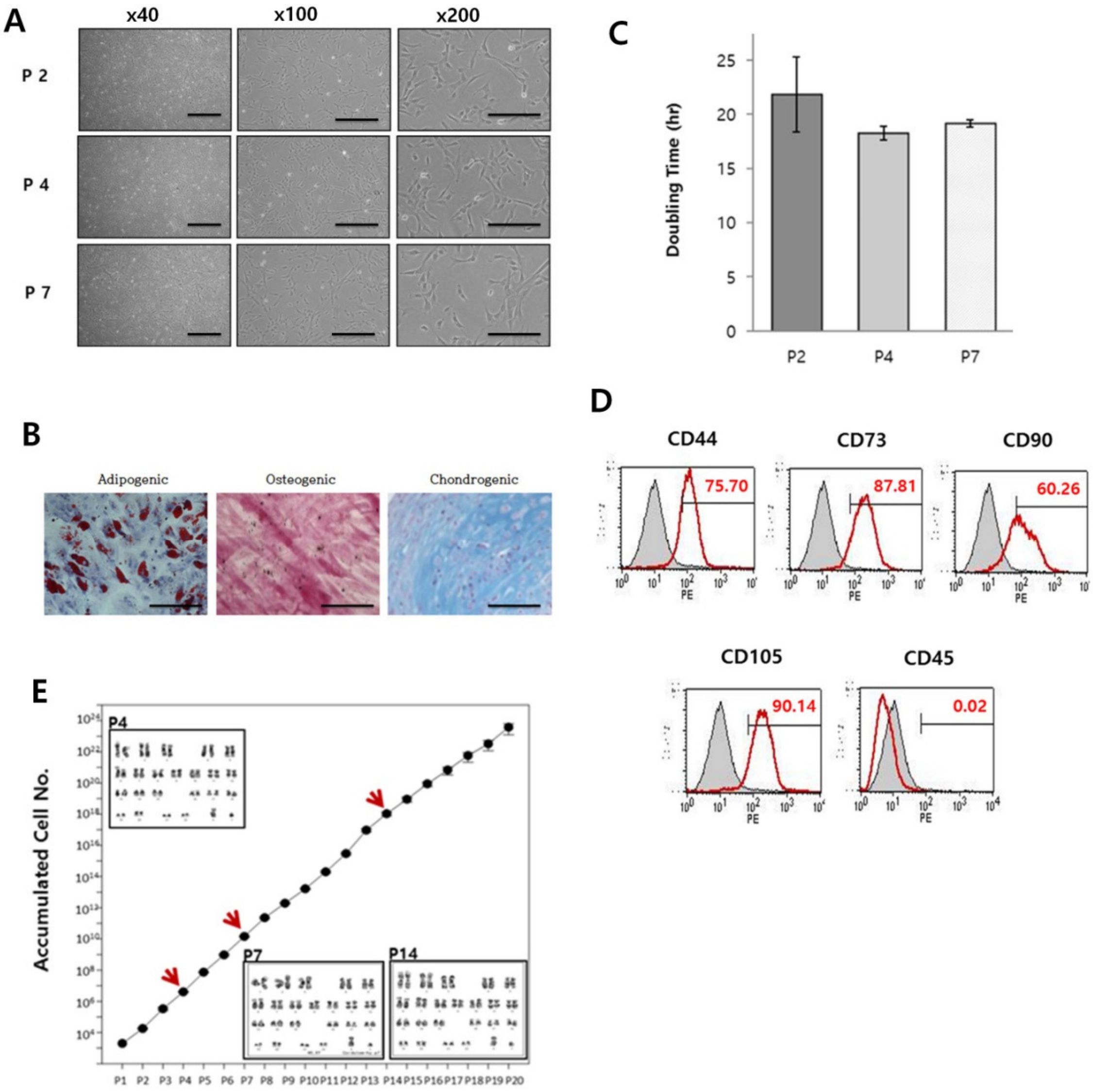

Figure S1. (A) Morphology of WJ-MSCs in the second, fourth, and seventh generation. P2, second generation; P4; fourth generation; P7; seventh generation (lens:×40,×100 and ×200 respectively). (B) Cellular multipotent differentiation of WJ-MSCs. Following adipogenic induction for 14 days, WJ-MSCs were positive for Oil red-O staining. Following osteogenic and chondrogenic induction for 14 days, WJ-MSCs were positive for alizarin red staining and alcian blue staining, respectively. Scale bars, 10 μm. (C) cell population doubling time. (D) flow cytometric analysis of cell surface markers of WJ-MSCs at P7. (E) chromosome karyotype analysis of the cultured WJ-MSCs.
